# Outcomes of Patients with Acute Retinal Necrosis (ARN) presenting to a Canadian tertiary care centre: a 5-year retrospective cohort study

**DOI:** 10.1186/s12348-026-00608-8

**Published:** 2026-06-11

**Authors:** Cody Lo, Jeffrey M. Mah, Carol Tadrous, Peter Greenstreet, Hong-An Nguyen, Chloe C. Gottlieb

**Affiliations:** 1 Department of Ophthalmology, Faculty of Medicine, University of Ottawa, Box 307 , Ottawa, ON K1H 8L6 Canada; 2 The Ottawa Hospital Research Institute (OHRI), University of Ottawa Eye Institute, Ottawa, ON Canada; 3 Department of Ophthalmology and Vision Sciences, Faculty of Medicine, St. Michael’s Hospital, University of Toronto, Toronto, ON Canada; 4 Department of Ophthalmology and Visual Sciences, Faculty of Medicine, University of British Columbia, Vancouver, BC Canada; 5 Ottawa Methods Centre, The Ottawa Hospital, Ottawa, ON Canada

**Keywords:** Uveitis, Herpes, Acute retinal necrosis

## Abstract

**Purpose:**

To describe the contemporary management and outcomes of patients with acute retinal necrosis (ARN) at a tertiary Canadian hospital over a 5-year period.

**Methods:**

This was a retrospective cohort study of patients who presented to The Ottawa Hospital (Ottawa, Canada) and were newly diagnosed with ARN between January 2019 and July 2024. Data regarding demographics, clinical presentation, investigations, treatment, and outcomes were collected. The primary outcome was retinal detachment. Secondary outcomes were severe vision loss and other complications associated with ARN. Statistical analyses were performed to identify factors associated with develop of the primary and secondary outcomes.

**Results:**

A total of 16 eyes amongst 14 patients met inclusion criteria. The mean age at presentation was 56 years old, with 57% male. 5 patients (36%) had a known history of previous systemic herpetic infection. The most common exam findings on presentation were anterior chamber cell (94%), keratitic precipitates (88%), vitritis (94%), and arteritis (75%). Aqueous humor serology was positive for 96% of eyes (56% for VZV and 40% for HSV). Eyes received an average of 5.8 injections (range 1 to 17) of intravitreal antiviral therapy. Corticosteroids were administered in 13 patients (81%). Prophylactic laser retinopexy was used in 1 eye (6%) and pars plana vitrectomy was done for vitreous opacities in 1 eye (6%). During a mean follow-up of 424 days, 44% of eyes developed retinal detachment. The average time from symptom onset to diagnosis of retinal detachment was 105 days (range 14 to 180). Half of eyes had a final visual acuity worse than 20/200. The most common complications were cataract (38%) and recurrent anterior uveitis (31%). Initial BCVA of worse than 20/400 was significantly associated with an outcome of severe vision loss. No associations were found for development of retinal detachment.

**Conclusion:**

Our cohort demonstrates the range of presentations and outcomes in the management of ARN. While early recognition and systemic antivirals remain essential, our centre uses a larger number of intravitreal antiviral injections with less prophylactic laser or vitrectomy to achieve similar outcomes as other cohorts. This study highlights the value of standardized protocols for recognition and treatment of ARN.

**Supplementary Information:**

The online version contains supplementary material available at 10.1186/s12348-026-00608-8.

## Introduction

Acute retinal necrosis (ARN) is a syndrome classically consisting of vitritis, retinal vasculitis and peripheral necrotizing retinitis [1, 2]. It is caused by a viral infection, typically with varicella zoster or herpes simplex virus, but in rare cases cytomegalovirus or Epstein-Barr virus [3, 4]. While it is rare with an incidence of approximately 0.5 cases per million population per year, it can result in severe vision loss when it occurs [3, 5]. Nearly half (48%) of affected eyes have a visual acuity worse than 20/200 at 6 months [3]. Retinal detachment is the most common cause of severe vision loss and occurs approximately in 20% to 73% of treated eyes [6, 7]. Additional morbidity can occur due to contralateral eye involvement with bilateral ARN occurring in up to 70% of untreated patients [8].

Owing to the rarity of ARN, the evidence guiding its management is relatively limited [9].

There is a consensus in the literature that systemic antivirals should be started immediately for the initial treatment of ARN and prevention of fellow eye involvement [9]. Either intravenous acyclovir or oral valacyclovir can be used as there are comparable plasma levels and rates of regression of retinitis with both.^9 10^ There is early evidence supporting the use of adjunctive intravitreal antivirals, however their use is not universal [11, 12]. Additionally, some have advocated for the use of prophylactic laser retinopexy or early pars plana vitrectomy before retinal detachment to reduce the risk of secondary retinal detachment, however the benefit of these interventions remains unproven [6, 10, 13]. A challenge in ARN management is many of aspects of treatment are based on level III evidence which includes descriptive or retrospective comparative studies [9]. ARN is difficult to study given its rarity, so the largest studies are typically retrospective and lack standardization in how patients are managed [14]. In addition to lack of consensus in the literature, heterogeneity in ARN management can stem from multiple logistical factors including delay in initial diagnosis, availability of subspecialist expertise, and local practice patterns.

The primary objective of this study was to describe the contemporary management and outcomes patients with ARN at a tertiary Canadian hospital over a 5-year period and to identify factors associated with poor outcomes. Amongst the uveitis subspecialists at our centre, we have adopted a consistent approach to ARN management. Additionally, our centre encompasses all the uveitis subspecialists for a large catchment area of Canada which likely reduces the risk of referral bias. Finally, our centre provides emergency ophthalmology coverage for the entire region and there is an expedited referral pathway for patients with emergent uveitis pathology. Therefore, we feel our cohort offers a unique perspective into the outcomes of ARN when managed with a standardized institutional protocol (Appendix A).

## Materials and methods

### Study design

This was a retrospective cohort study of patients who presented to The Ottawa Hospital (Ottawa, Canada) and were newly diagnosed with ARN between January 2019 and July 2024. Data was collected until August 2025. This study was approved by the Ottawa Health Science Network Research Ethics Board (Protocol 2023044–01 H).

### Study population

Patients were identified through a keyword search for “acute retinal necrosis” of the electronic medical record. Charts were screened by three authors (CL, JMM, CT) to confirm the diagnosis. ARN was diagnosed clinically based on the definition proposed by the American Uveitis Society which includes the following requirements: “focal, well-demarcated areas of retinal necrosis located in the peripheral retina (outside of the major temporal vascular arcades); rapid, circumferential progression of necrosis (if antiviral therapy has not been administered); evidence of occlusive vasculopathy; and a prominent inflammatory reaction in the vitreous, and anterior chamber.”^1^ Charts were excluded if an alternative diagnosis was equally or more likely after review (e.g. CMV retinitis).

### Covariates

Charts were reviewed for collection of the following data:

1. Baseline demographics: sex, age, immunosuppression status, and unilateral or bilateral involvement;
2. Clinical presentation: previous eye disease, previous herpetic disease, symptoms, symptom duration, best corrected visual acuity (BCVA), intraocular pressure, lens status, and other clinical signs;
3. Investigations: anterior chamber or vitreous sampling for viral testing.
4. Treatment: systemic antivirals, intravitreal antivirals, systemic corticosteroids, prophylactic laser retinopexy, or early prophylactic pars plana vitrectomy before retinal detachment.

### Outcomes

The primary outcome was retinal detachment. Secondary outcomes were fellow eye involvement, recurrent anterior uveitis, cataract, cystoid macular edema, optic neuropathy, and severe vision loss. Severe vision loss was defined as Snellen visual acuity worse than 20/200, based on previous studies [14–16].

### Data analysis

Means and standard deviations were used to describe normally distributed variables. Medians and interquartile ranges were used for variables with a non-normal distribution. BCVA was converted to logMAR and treated as a continuous variable. Comparisons were performed using non-parametric tests such as the Fisher exact test for categorical variables or the Mann Whitney U test for continuous variables using R version 4.4.2 for Windows.

## Results

A total of 16 eyes amongst 14 patients were included. The average follow up was 424 days with a range of 13 to 1183 days.

### Baseline demographics and clinical presentation

**Fig. 1:**
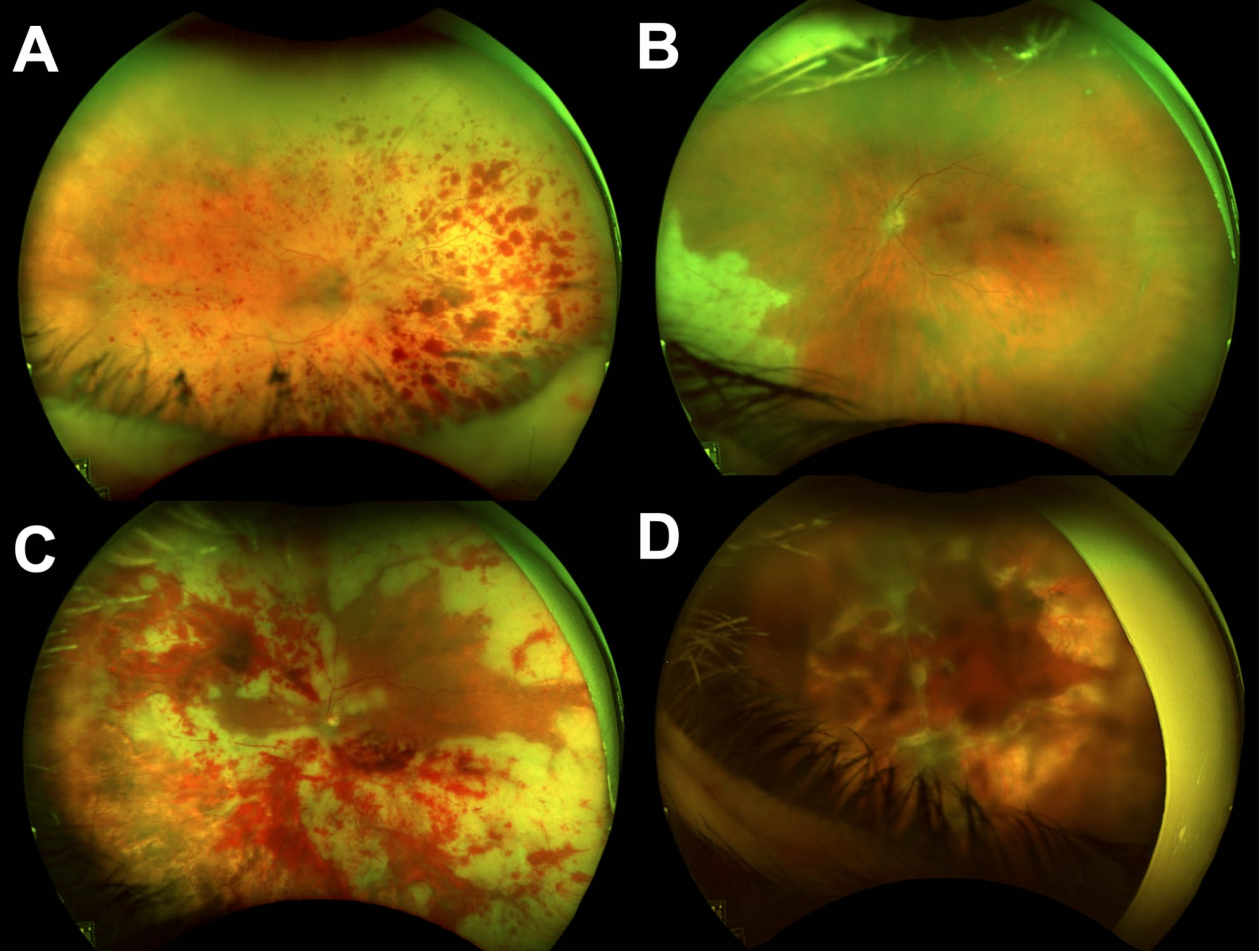
Representative pseudo color images of patients presenting with acute retinal necrosis (ARN). (**A**) Patient 7 on presentation with retinal whitening most prominent nasally, vascular sheathing, peripheral vessel sclerosis, and diffuse retinal hemorrhages (**B**) Patient 5 on presentation with a dense focal area of retinal whitening inferonasal, vascular sheathing, and optic disc edema (**C**) Patient 4 on presentation with 360 degree large patches of retinal whitening with involvement of both the optic nerve and macula (**D**) Patient 4 two years after pars plana vitrectomy and silicon oil for retinal detachment. There is anatomic attachment of the retina despite multiple fibrous bands and media opacities. This patient had a final visual acuity of hand motions only

The mean age was 56 years (SD 21 years). The majority of patients were male (57%) and presented with unilateral disease (88%). Five patients (36%) had a history of systemic herpetic disease. No patients have previous ocular herpetic disease. The mean time from symptom onset to assessment by an ophthalmologist was 13 days (range 0 to 42). The mean BCVA on presentation was 1.6 logMAR (20/800 Snellen acuity, SD 1.3). Table 1 describes the baseline demographics and clinical presentation. Fundus images of all includes eye were confirmed to have features of ARN as described by the SUN criteria (Fig. 1 and Appendix B). Only 38% of eyes in our cohort had a correct initial diagnosis of ARN with other initial diagnoses including anterior uveitis (38%) or another posterior uveitis (19%).

**Table 1:** Characteristics of eyes presenting with acute retinal necrosis (ARN)

	*n* (%)	*N*
	8 (57%)	14
**Male gender** ^1^		
• Age at diagnosis, years^1^ (mean (range))	56 (22, 86)	14
• Immunosuppressive medication use	6 (38%)	16
**Medical comorbidities**		
• Rheumatoid arthritis	1 (6%)	16
• Systemic lupus erythematosus	1 (6%)	16
**Previous herpetic infection**		
• Herpes simplex virus encephalitis	3 (19%)	16
• Varicella zoster virus dermatitis^2^	3 (19%)	16
**Specialist involvement**		
• Vitreoretinal specialist	4 (25%)	16
• Uveitis specialist	2 (13%)	16
• Both	10 (62%)	16
**Presenting symptoms**		
• Reduced vision	14 (88%)	16
• Pain	11 (69%)	16
• Eye redness	10 (63%)	16
• Photophobia	5 (31%)	16
• Floaters	3 (19%)	16
• Photopsias	0 (0%)	16
**Initial eye care provider**		
• Ophthalmologist	11 (69%)	16
• Optometrist	5 (31%)	16
**Initial diagnosis**		
• Acute retinal necrosis	6 (38%)	16
• Anterior uveitis	6 (38%)	16
• Posterior uveitis (not including ARN)	3 (19%)	16
• Retinal vasculitis (not including ARN)	1 (6%)	16
**Initial best corrected visual acuity**		
• Better or equal to 20/50	5 (31%)	16
• 20/50 to 20/200	2 (13%)	16
• Worse or equal to 20/200	9 (56%)	16
**Initial IOP**		
• Mean (mmHg) (range)	16 (8, 36)	16
• > 21 mmHg or on intraocular pressure lowering agents	4 (25%)	16
**Initial exam findings**		
• Keratitis	0 (0%)	16
• Anterior chamber inflammation	15 (94%)	16
• Keratitic precipitates (KP)	14 (88%)	16
• KP, granulomatous	4 (25%)	16
• KP, nongranulomatous	5 (31%)	16
• KP, non specific	5 (31%)	16
• Phakic	15 (94%)	16
• Vitritis	15 (94%)	16
• Arteritis	12 (75%)	16
• Macula involvement	8 (62%)	13^3^
• Optic nerve head involvement	11 (79%)	14^1^
Time from symptom onset to diagnosis, days (mean (range))	13 (0, 28)	16

1. Reported as per patient, two patients had both eyes included in the study

2. Two eyes were from same patient who had history of vesicular dermatitis on their leg a year prior to visual symptoms. The other patient had ipsilateral cranial nerve V1 dermatitis with onset of visual symptoms

3. Assessment of posterior segment findings were indeterminate in some images due to media opacities

### Investigations

All eyes underwent anterior chamber sampling for viral testing. The sample was tested for VZV in 100%, HSV-1 and HSV-2 in 94%, CMV in 81% and toxoplasmosis in 6% of cases. Nine eyes (56%) were positive for VZV and 6 eyes (40%) were positive for HSV-1. One eye was PCR-negative for all viruses tested but the other eye of this patient was positive for VZV. No eyes were positive for CMV or toxoplasmosis. Vitreous sampling was not performed on any eyes.

### Treatment

All patients received induction antiviral therapy with 10 mg/kg of systemic intravenous acyclovir every 8 h or 2 g of valacyclovir orally in three times a day for 7–10 consecutive days. Maintenance oral antiviral therapy was used on average for 10.8 weeks (range 8 to 12 weeks, *n* = 8 patients). All patients also received a 2.0 mg/0.1 ml intravitreal injection of ganciclovir based on local hospital protocol (Appendix A). Patients received intravitreal injections twice a week as the induction treatment until retinitis stabilized, at which time the frequency decreased to approximately once a week until retinitis was considered inactive (Appendix A). Seven patients (50%) were kept on indefinite prophylactic oral antivirals. The medication dose was adjusted for patients with renal insufficiency.

Eyes were treated by a fellowship trained vitreoretinal specialist in 25%, a fellowship trained uveitis specialist in 13%, and were co-treated by both in 63%. The mean time from diagnosis to initiation of oral antivirals was − 0.6 days (range − 1 to 1) as most patients were started on some form of treatment prior to getting referred to our tertiary centre. All eyes were treated with systemic antiviral medication on presentation. One patient with bilateral ARN received treatment with intravenous acyclovir. Thirteen patients (93%) received oral treatment with 10 (77%) receiving valacyclovir and 5 (38%) receiving famciclovir. All eyes were treated with intravitreal antivirals. Most eyes (88%) received a regimen of intravitreal antivirals that included both ganciclovir and foscarnet while 2 patients received only ganciclovir. The mean time from presentation to initiation of intravitreal antivirals was 0.6 days (SD 0.8). The mean number of intravitreal antiviral injections was 5.88 (range 1 to 17). Adjunct oral corticosteroids were used in 10 patients (71%) with a dose of 1 mg/kg up to 60 mg daily was started approximately 48–72 h after the initiation of antiviral treatment. On average the treatment dose of steroids was used for 1.7 weeks (range 2 days to 5 weeks, *n* = 9 patients). Intravenous steroids were used in 1 patient (7%) with 1000 mg of methylprednisolone given for 3 days followed by tapering doses of oral prednisone. Systemic steroids were tapered based on clinical response and total length of treatment including taper was on average 5.8 weeks (range 1 to 11 weeks, *n* = 5 patients). The mean time from initiation of antivirals to initiation of corticosteroids was 5.4 days (SD 1.5). Prophylactic laser was performed in 1 eye and early prophylactic PPV (not for retinal detachment) was performed in 1 eye. Seven patients (50%) were prescribed indefinite prophylactic oral antiviral medication. Table 2 describes the investigations and treatment.

**Table 2:** Investigations and management of eyes with acute retinal necrosis presenting to a tertiary care centre

	*n* (%)	*N*
**Investigations completed**		
Aqueous humor sample	16 (100%)	16
• Varicella zoster virus	9 (56%)	16
• Herpes simplex virus	6 (40%)	15
• Cytomegalovirus	0 (0%)	13
• Toxoplasmosis	0 (0%)	1
Vitreous humor sample	0 (0%)	16
Treatments used		
**Antivirals**		
Systemic Antivirals	16 (100%)	16
• Oral	14 (87%)	16
• Intravenous	2 (13%)	16
Intravitreal	16 (100%)	16
• Ganciclovir	16 (100%)	16
• Foscarnet	14 (87%)	16
Number of intravitreal antiviral injections (mean (range))	5.8 (1, 17)	16
Time from **symptom onset** to initiation of **oral antivirals**, days (mean (range))	13 (0, 42)	16
Time from **symptom onset** to initiation of **intravitreal antivirals**, days (mean (range))	14 (0, 42)	16
**Systemic steroids**		
• Any systemic steroids	13 (81%)	16
• Prednisone (oral)	11 (85%)	13
• Methylprednisolone (intravenous)	2 (15%)	13
Time from **symptom onset** to initiation of **adjunct systemic steroids**, days (mean (range))	19 (0, 50)	13
Time from **initiation of antivirals** to initiation of **adjunct systemic steroids**, days (mean (range))	5 (-1, 15)^1^	13
**Prophylactic treatments**		
Prophylactic laser performed	1 (6%)	16
Time from **diagnosis** to initiation of **prophylactic laser**, days (mean (range))	49 (49, 49)	1
Prophylactic oral antivirals	8 (50%)	16

1. Some eyes received treatment prior to diagnoses being made

### Outcomes

Table 3 describes the final outcomes of eyes presenting with ARN. Retinal detachment occurred in 7 (44%) eyes. Retinal detachment was diagnosed on average 105 days from symptom onset (range 14 to 180 days). Two eyes (29%) were treated with barrier laser only whereas the other 5 (71%) eyes underwent pars plana vitrectomy, endolaser, and silicon oil insertion. All patients undergoing vitrectomy had anatomic attachment of the retina whereas those treated with laser only did not have progression of the retinal detachment. Five (71%) eyes following retinal detachment had a final visual acuity worse than 20/400 (range 20/80 to NLP). No clinical characteristics were found to be statistically significant with development of retinal detachment (Appendix C).

**Table 3:** Outcomes of eyes with acute retinal necrosis presenting to a tertiary care centre

	*n* (%)	*N*
	7 (44%)	16
**Retinal detachment**		
• Time from **symptom onset** to diagnosis of **retinal detachment**, days (mean (range))	105 (14, 180)	16
• Time from **diagnosis** to diagnosis of **retinal detachment**, days (mean (range))	88 (0, 165)	16
**Other complications**		
• Cataract	6 (38%)	16
• Recurrent anterior uveitis	5 (31%)	16
• Elevated intraocular pressure	2 (13%)	16
• Epiretinal membrane	2 (13%)	16
• Cystoid macular edema	1 (6%)	16
**Final best corrected visual acuity**		
• Severe vision loss (worse than 20/200)	8 (50%)	16
• Better or equal to 20/50	3 (19%)	16
• 20/50 to 20/200	5 (31%)	16
• Worse than 20/200 to counting fingers	0 (0%)	16
• Hand motion	3 (19%)	16
• Light perception	2 (13%)	16
• No light perception	3 (19%)	16

Amongst eyes presenting with unilateral ARN, one (7.7%) developed fellow eye involvement 32 days after symptom onset. 1 eye (6%) developed cystoid macular edema. Most eyes (11, 79%) had optic nerve head involvement defined as optic disc edema or RAPD on presentation with or without retinitis involving the peripapillary retina. No patients were diagnosed with optic neuropathy following resolution. Other complications in the cohort include cataract (38%), and recurrent anterior uveitis (25%). Severe vision loss occurred in 8 eyes (50%) by the time of the last follow-up. Initial BCVA of worse than 20/400 was significantly associated with an outcome of severe vision loss (Appendix C).

## Discussion

In this retrospective cohort study, we describe the clinical presentation, management, and outcomes of patients with ARN presenting to a Canadian tertiary care center over a five-year period. During this time, our institution followed a standardized management protocol including frequent intravitreal antiviral injections (Appendix A). This is the largest Canadian cohort of ARN patients reported to date [17]. ARN remains a rare but vision-threatening condition, and timely diagnosis and treatment are critical to preserving vision. Our findings highlight the spectrum of presenting features as well as contemporary management and outcomes.

Incidence of ARN has been estimated to be around 0.5 cases per million population per year based on multiple prospective database studies from the UK.^3,5^ An Australian study also reported an incidence of 0.5 cases per million population per year [18]. The approximate incidence in our Canadian centre is slightly higher than these studies at 1.6 cases per million population per year over this recent 5 year interval with a catchment population of approximately 2 million. The incidence at our Canadian centre is in keeping with another single centre observational study in the US which showed an average of 3.7 cases/year [14].

The demographic and clinical features observed in our cohort are broadly consistent with prior reports of ARN, which often emphasize the predominance in middle-aged individuals without a gender predilection [4, 5]. Similar to other studies, our cohort was primarily immunocompetent individuals, though immunosuppression is a known risk factor [19]. An observation in our cohort that has been infrequently reported in prior studies was that 38% of eyes were receiving low-dose oral prednisone at symptom onset for unrelated autoimmune conditions, such as systemic lupus erythematosus or polymyalgia rheumatica. While ARN is typically considered a disease of immunocompetent individuals there have been speculation about whether the spectrum of herpetic disease reactivation is influenced by the immune state of the host [20]. Another study noted 12 of 22 patients with ARN had some mild immune “dysregulation” such as systemic malignancy, on medication for rheumatoid arthritis, recent course of prednisone for herpes zoster oticus, or diabetes mellitus [18]. Anecdotally, both patients in our cohort with bilateral presentation of ARN were on oral prednisone. While speculative, this could have implications in counselling patients about their risk of ARN with previous severe herpetic disease such as encephalitis and subsequent long-term use of medications like prednisone, or when diagnosed with inflammatory conditions later in life.

While the majority of patients did not report a previous herpetic disease episode, the most common previous manifestation of HSV was encephalitis in 3 patients (21%) which has been shown in multiple previous studies ranging from 13%-23.9% [3, 21, 22]. In our review, there are no studies published showing the prevalence of ARN amongst patients with previous HSV encephalitis. While the exact mechanism of this association is unknown, it is hypothesised that ARN occurring after herpes simplex encephalitis is due to reactivation of latent virus with anterograde axonal spread from the trigeminal ganglion to the retina. The most common manifestation of VZV was dermatitis in 19% of eyes. A series of 7 patients with ARN following dermatitis has been reported with the length of time between the skin infection and ARN ranging from 5 days to 3 months [23]. Our cohort demonstrated a highly variable time from symptom onset to both presentation and diagnosis. This is likely due to early symptoms and signs possibly being mild and non-specific.

The majority of eyes were PCR positive for viral DNA in the aqueous with VZV being the most common pathogen (56%) followed by HSV (40%). This finding is similar to other cohorts which report 66.7% − 69.6% VZV positivity [24, 25]. Only one eye did not have a positive PCR aqueous sample in our cohort. This case was one of the patients with bilateral ARN presentation and the other more involved eye was PCR positive for VZV. So ultimately lack of PCR positivity did not change management in this case. These results demonstrate that aqueous humor PCR can be a highly sensitive method for confirming the diagnosis which can be helpful in atypical or early cases to institute treatment prior to vision loss. The mean time of 15 days from symptom onset to initiation of investigation in our series also reflects ongoing challenges in timely initiation of management for ARN, particularly when in the early stages the signs and symptoms can be non-specific and difficult to differentiate from other less urgent types of infectious or non-infectious uveitis.

The time from symptom onset to various management timepoints was similar in a retrospective study from the US where it took on average 12 days from symptom onset to first visit where investigations were started [14]. Another study of 39 patients in Switzerland found a mean time from symptom onset to treatment of 15 days with worse initial vision and longer time to treatment being significantly correlated with worse final visual outcome [26]. Our study did not find a similar statistically significant association but our cohort is less than half the size and thus may be underpowered. Optic nerve involvement is thought to be associated with severe vision loss due to optic neuropathy. Our cohort had a high level (79%) of optic nerve involvement which is also higher than previous cohorts reporting 44% [14]. This difference may be in part due to inclusion of any eyes with optic disc edema as opposed to other studies which require evidence of optic nerve dysfunction which likely represents more advanced involvement [14]. Despite this high prevalence optic nerve involvement was not associated with severe vision loss or retinal detachment in our study.

Antiviral therapy remains the cornerstone of treatment, with adjunctive corticosteroids and surgical interventions considered in select cases. Our series demonstrated a higher use of oral antivirals as the main systemic coverage compared to some previously published cohorts where 75.6% of patients received intravenous antivirals [3]. These results provide further support to the claim that oral or intravenous antiviral therapy are effective in treating ARN [3]. However, in our experience oral antiviral therapy is more convenient for patients particularly at our tertiary referral centre when patients do not have easy access to an outpatient infusion centre. Our institution can compound both ganciclovir and foscarnet as intravitreal injections so decision to use one over another was determined by the physician. There lacks evidence whether efficacy and intraocular penetration for a particular antiviral varies between systemic versus intravitreal administration [27]. Pharmacokinetic studies in rabbits comparing intravitreal concentration of ganciclovir vs. foscarnet demonstrated higher peak levels and longer sustained concentration above therapeutic levels at multiple time points for ganciclovir [28]. Foscarnet can be beneficial for its different side effect profile and to treat disease resistant to ganciclovir [27].

There has been discussion in the literature about whether intravitreal antiviral reduces the incidence of retinal detachment [11, 12]. One comparative cohort study of 24 patients with ARN showed a combination of systemic and intravitreal therapy were more likely to gain two or more lines of visual acuity and showed decreased incidences of severe visual loss and retinal detachment compared to systemic antiviral alone [12]. Another study found a 30% lower rate of retinal detachment (53.6% from 75.0%) when using intravitreal foscarnet for ARN associated with VZV [11]. Our retinal detachment rate of 44% is slightly lower than previous studies which may be related to all patients receiving multiple intravitreal antiviral injections as part of a local protocol [11]. We followed patients on average for over a year in this study which is longer than most published cohorts and should capture the majority of retinal detachments [29]. It is possible that early intravitreal antiviral administration may prolong the time to retinal detachment. Our cohort seems to exhibit a longer mean time from onset of symptoms to retinal detachment with an average of 105 days. This is in contrast to other cohorts where no intravitreal antivirals were given and report an average of 63.3 days before retinal detachment [24]. Despite multiple cohort studies suggesting that intravitreal antiviral injections may reduce the rate of retinal detachment, this association was not found to be statistically significant in two meta analyses of 1811 and 963 participants respectively [27, 30]. However a limitation of this finding is that the dosing of intravitreal antivirals amongst the included studies was heterogenous ranging from only a single injection to multiple injections per week. Frequent injection dosing as done in our cohort may be required to confer the protective effects for retinal detachment. The one patient who received prophylactic laser in our study was in the context of vitrectomy for significant vitreous opacities and did not subsequently develop retinal detachment. However from this study we are not able to draw any conclusions about the value of prophylactic laser which remains to be debated in the literature [9]. The majority of studies advocating for prophylactic laser to prevent retinal detachment are observational in nature [7]. Our observation of 50% of patients having a final visual outcome of 20/200 or worse is in keeping with previous studies showing 54% of 104 eyes followed for more than 1 year amongst 9 different centres [13].

The data exploring early pars plana vitrectomy to prevent retinal detachment and improve visual outcomes has been mixed. Despite only one patient receiving early pars plana vitrectomy in our cohort, we report similar final visual outcomes and retinal detachment rates as previous studies where the rate of early vitrectomy was higher. One study of 30 eyes found 90% of patients without vitrectomy developed retinal detachment compared to 40% in the vitrectomy group [6]. However there are studies showing the opposite conclusion such as a large study of 104 eyes with ARN which found a retinal detachment rate of 42% in the early vitrectomy group compared with 25% in the observation group with no visual benefits [13]. Another challenge in determining the relationship between vitrectomy and preventing retinal detachment or severe vision loss is that often observational studies include multiple indications for vitrectomy such as retinal detachment on presentation and dense visually significant vitritis [16]. As discussed by Choi et al., this bias can affect the positive correlation between early vitrectomy and the risk of vision loss. While not reported in the literature, vitrectomy in eyes following ARN could be at higher risk of developing iatrogenic breaks or other post-vitrectomy complications. Additionally, there have been cases of ARN occurring days after vitrectomy, including for open globe repair [31]. In our study, the one patient who received prophylactic laser and vitrectomy was symptomatic from persistent vitreous opacities. So, the vitrectomy in this case was partially for visual rehabilitation in addition to potentially reducing risk of subsequent retinal detachment. This heterogeneity amongst the various prophylactic treatments is likely confounded by the concurrent use of other treatment modalities. These inconsistencies in the literature underscore the lack of consensus and the need for prospective trials to clarify optimal treatment algorithms.

The present study has several limitations inherent to its retrospective design. First, the rarity of ARN limited our sample size, which restricts statistical power and generalizability. We attempted to compare the clinical characteristics and management of patients with severe vision loss or retinal detachment using nonparametric testing but suspect our sample size was underpowered to determine statistical significance. Second, incomplete records over time may have introduced bias. This was minimized by use of a standard ARN protocol at our centre and many of the diagnostics were available for retrospective review for specific features of interest. Third, the absence of a control group precludes direct comparison of different treatment strategies. Finally, as a single-center study, referral bias and local practice patterns may have influenced our results. Our single center includes all the uveitis and vitreoretinal subspecialists for a large geographic area in Canada and thus is likely less susceptible to referral bias but patients in this catchment area may not be reflective of the broader population.

Future research should focus on multicenter prospective studies to increase cohort sizes, better define prognostic factors, and optimize treatment protocols. Advances in molecular diagnostics, such as polymerase chain reaction (PCR) analysis of intraocular fluids, may help reduce diagnostic delays and allow for more targeted antiviral therapy. While consensus statements from governing organizations have been helpful, published guidelines would help reduce variability in practice [9]. Standardized use of adjunctive treatments, including corticosteroids, anticoagulation, and prophylactic laser, would help compare outcomes across different centres. Collaborative registries may be particularly valuable in overcoming the rarity of ARN and in generating higher-quality evidence to inform clinical care.

Our five-year experience highlights both the progress and the persistent challenges in the management of ARN. While early recognition and systemic antivirals remain essential, our centre uses a larger number of intravitreal antiviral injections with less prophylactic laser or vitrectomy to achieve similar visual outcomes as other cohorts. Broader collaboration, earlier detection, and standardized treatment strategies will be key to improving outcomes in this vision-threatening disease.

## Supplementary Information

Below is the link to the electronic supplementary material.

Supplementary Material 1

## Data Availability

The datasets used and/or analysed during the current study are available from the corresponding author on reasonable request.
