# Supplementary material for: Outcomes of Patients with Acute Retinal Necrosis (ARN) presenting to a Canadian tertiary care centre: a 5-year retrospective cohort study
Source: J Ophthalmic Inflamm Infect. 2026 Jun 11;16:46. doi: 10.1186/s12348-026-00608-8 (PMC13478040; doi:10.1186/s12348-026-00608-8)
Supplement: Supplementary file 1 [file 12348_2026_608_MOESM1_ESM.docx]

**Appendices**

**Appendix A. Description of institutional treatment for acute retinal necrosis (ARN)**

To be used under supervision of a vitreoretinal and/or uveitis subspecialist

**1. Clinical Objectives**

- Achieve rapid halt of viral replication and retinal necrosis.
- Prevent fellow-eye involvement (present in up to 30% of untreated cases).
- Minimize the risk of rhegmatogenous retinal detachment (RRD).

**2. Diagnostic Criteria**

The diagnosis of ARN requires Peripheral Necrotizing Retinitis AND at least one of the following:

- Positive PCR for Herpes Simplex Virus (HSV-1 or HSV-2) or Varicella-Zoster Virus (VZV) from an intraocular fluid specimen (aqueous or vitreous).
- Characteristic clinical appearance, defined by:
  - Circumferential or confluent retinitis.
  - Evidence of retinal vascular inflammation (e.g., periarteritis or occlusive vasculopathy).
  - Greater than minimal vitritis (unless the patient is immunocompromised).

**3. Standard Investigations**

Upon suspicion of ARN, the following investigations should be initiated immediately:

- Ocular Diagnostics
  - Intraocular Fluid Analysis: Diagnostic AC paracentesis or vitreous tap for Viral PCR (such as HSV-1, HSV-2, VZV, CMV depending on volume of sample acquired).
  - *Note: Treatment must not be delayed for PCR results.*
- Imaging:
  - Ultra-widefield fundus imaging (e.g., Optos) for documentation and monitoring of "leading edge" progression.
  - Optical Coherence Tomography (OCT) of the macula and optic nerve to assess for edema or involvement. *Note: will depend on media clarity*
- Other tests to consider:
  - Fluorescein Angiography (FA) if occlusive vasculitis or ischemia requires quantification.
  - Systemic workup to rule out differentials and assess risk of treatment side effects
    - Baseline Labs: CBC, Electrolytes, Urea, and Creatinine (essential for antiviral dosing).
    - Rule-out Panel: HIV (p24 antigen/antibody), Syphilis (RPR/TPPA), QuantiFERON-Gold (TB), and Toxoplasma serology (IgG/IgM).

**4. Treatment Protocol**

Phase I: Induction (Days 1 to 10–14)

All patients should receive combination antiviral therapy (Systemic + Intravitreal) at the time of diagnosis.

A. Intravitreal Therapy (Local Control)

- Agents: Foscarnet (2.4 mg/0.1 mL) or Ganciclovir (2.0 mg/0.1 mL).
- Frequency: Twice weekly until retinitis is consolidated/inactive.

B. Systemic Therapy (Systemic Control & Fellow Eye Protection)

- Option 1 (Inpatient/Severe): IV Acyclovir 10 mg/kg to 15 mg/kg every 8 hours for 7 to 14 days.
- Option 2 (Outpatient/Moderate): Oral Valacyclovir 2 g three times daily (6 g total daily) for 10 to 14 days.
- *Note:* Adjust all dosages based on renal clearance (CrCl).

Phase II: Maintenance (Weeks 2 to 12+)

- Transition to maintenance oral therapy once retinitis shows signs of regression (blurring of margins, thinning of necrotic areas).
- Standard Regimen: Oral Valacyclovir 1 g three times daily for 6 weeks but can alter based on treatment response
- Taper/Long-term: Consider reduction to 1 g daily indefinitely depending on clinical response and immune status.

**5. Adjunctive Management**

*Note: use as needed*

Corticosteroids

- Systemic: Oral Prednisone (0.5 mg/kg to 1 mg/kg daily).
- Timing: Initiate 24 to 48 hours after the start of antiviral therapy.
- Purpose: To manage vitritis and reduce the risk of secondary tractional complications.

Surgical Intervention

- Prophylactic Laser: Consider barrier laser photocoagulation posterior to the areas of necrosis once media clears.
- Pars Plana Vitrectomy (PPV): Indicated for retinal detachment, dense vitritis obscuring the view, or in selected cases of rapidly progressive necrosis (early PPV).

**6. Monitoring Schedule**

- Induction: Clinical exam every 2 to 3 days (coinciding with intravitreal injections).
- Maintenance: Weekly for 1 month, then every 2 to 4 weeks until the 3-month mark.
- Dilation: Mandatory 360-degree depressed exam at every visit to monitor for retinal breaks at the junction of necrotic and healthy retina.

**Appendix B. Qualitative description of fundus exam for patients presenting with acute retinal necrosis**

| Patient number | Description |
| --- | --- |
| Patient 1 | Multiple foci of peripheral retinal whitening in the inferior 180 degrees |
| Patient 2 | Superior and temporal retinal whitening |
| Patient 3 | Inferotemporal retinal whitening, serous elevation inferiorly and superiorly |
| Patient 4 (eye 1) | 360 degree large patches of retinal whitening |
| Patient 4 (eye 2) | Superotemporal patch of hemorrhage |
| Patient 5 | Inferonasal peripheral retinal whitening |
| Patient 6 | 360 degree patchy eripheral retinal whitening |
| Patient 7 (eye 1) | Vascular sheathing, peripheral vessel sclerosis, retinal hemorrhages |
| Patient 7 (eye 2) | Vascular sheathing, peripheral vessel sclerosis, retinal hemorrhages |
| Patient 8 | 360 degree patchy peripheral retinal whitening |
| Patient 9 | Vascular sheathing, 360 degree retinal whitening |
| Patient 10 | Multiple foci of peripheral retinal whitening 360 degrees |
| Patient 11 | Multiple foci of peripheral retinal whitening 360 degrees, temporal retinal detachment |
| Patient 12 | Inferotemporal peripheral retinal whitening |
| Patient 13 | Multiple foci of peripheral retinal whitening superior, inferior and temporal |
| Patient 14 | Retinal whitening temporal |

**Appendix C. Comparison of patients with severe vision loss or retinal detachment following acute retinal necrosis**

|  | Severe vision loss | | | Retinal detachment | | |
| --- | --- | --- | --- | --- | --- | --- |
|  | Yes (n=8) | No (n=8) | P value | Yes (n=7) | No (n=9) | P value |
| Etiology | | | | | | |
| HSV | 4 | 2 | 0.61 | 2 | 4 | 0.63 |
| VZV | 4 | 6 |  | 5 | 5 |  |
| Different initial diagnosis | | | | | | |
| Yes | 6 | 4 | 0.61 | 4 | 6 | 1.00 |
| No | 2 | 4 |  | 3 | 3 |  |
| Initial best corrected visual acuity worse than 20/400 | | | | | | |
| Yes | 6 | 1 | 0.04 | 4 | 3 | 0.61 |
| No | 2 | 7 |  | 3 | 6 |  |
| Macula involvement | | | | | | |
| Yes | 5 | 3 | 0.41 | 3 | 5 | 0.82 |
| No | 1 | 4 |  | 2 | 3 |  |
| Indeterminate | 2 | 1 |  | 2 | 1 |  |
| Optic nerve head involvement | | | | | | |
| Yes | 7 | 4 | 0.20 | 5 | 6 | 1.00 |
| No | 0 | 3 |  | 1 | 2 |  |
| Indeterminate | 1 | 1 |  | 1 | 1 |  |
| Use of adjunctive oral steroids | | | | | | |
| Yes | 7 | 6 |  | 6 | 7 | 1.00 |
| Yes | 1 | 2 |  | 1 | 2 |  |
| Time to various management time points - Mean (SD), Median [IQR] | | | | | | |
| Onset of symptoms to diagnosis | 15.0 (11.6), 14 [10.3–14.0] | 11.4 (9.5), 9.5 [4.5–16.5] | 0.52 | 16.9 (11.6), 14.0 [14.0, 14.5] | 10.3 (9.03), 8 [5, 12] | 0.10 |
| Diagnosis to initiation of systemic antivirals | –0.13 (0.64), 0 [–0.25–0] | 0 (0), 0 [0–0] | 0.54 | 0 (0), 0 [0, 0] | -0.11 (0.60), 0 [0, 0] | 0.59 |
| Diagnosis to initiation of adjunctive oral steroids | 2.6 (4.2), 1 [–0.5–5.0] | 8.7 (5.3), 8.5 [7–12.3] | 0.07 | 5.17 (5.04), 1.25 [4.5, 7.75] | 5.57 (6.32), 7.0 [-0.5, 9.5] | 0.33 |
